# Differentials in prevalence and correlates of metabolic risk factors of non-communicable diseases among women in sub-Saharan Africa: evidence from 33 countries

**DOI:** 10.1186/s12889-018-6085-2

**Published:** 2018-10-11

**Authors:** Sanni Yaya, Michael Ekholuenetale, Ghose Bishwajit

**Affiliations:** 1 School of International Development and Global Studies, University of Ottawa, 120 University Private, Ottawa, ON K1N 6N5 Canada; 2 Department of Epidemiology and Medical Statistics, Faculty of Public Health, College of Medicine, University of Ibadan, Ibadan, Nigeria

**Keywords:** Non-communicable diseases, Hypertension, Obesity, Blood pressure, Risk factors

## Abstract

**Background:**

Even with the widespread recognition of non- communicable diseases (NCDs) in sub-Saharan Africa region, yet, sufficient evidence-based surveillance systems to confirm the prevalence and correlates of these diseases is lacking. In an attempt to understand the problem of NCDs in resource-constrained settings, this study was conducted to establish the pattern of the risk factors of NCDs in sub-Sahara Africa region.

**Methods:**

The current Demographic and Health Survey (DHS) data sets from 33 countries in sub-Sahara Africa region were used in this study. The individual woman component of DHS 2008–2016 was used. The outcome variables include anemia, hypertension and body mass index (underweight, overweight and obesity). BMI was categorized into; underweight (BMI < 18.5 kg/m^2^), normal (BMI 18.5–24.9 kg/m^2^), overweight (BMI 25.0–29.9 kg/m^2^) and obesity (BMI ≥30 kg/m^2^). Hemoglobin level: anemic < 12.0 g/dL (< 120 g/L) for women. Hypertension was defined as systolic blood pressure (SBP) ≥140 mmHg and/or diastolic blood pressure (DBP) ≥90 mmHg. Binary and multinomial logistic regression models were used to investigate the correlates of the variables.

**Results:**

The percentage of hypertension was highest among women in Lesotho with about 17.3% and lowest among women in Burundi (1.0%). Anemia was prevalent among sub-Saharan Africa women; where more than half of the women from several countries were anemic with Gabon (60.6%) reporting the highest prevalence. The percentage of obesity in sub-Saharan Africa showed that Lesotho (19.9%), Gabon (18.9%) and Ghana (15.6%) were the prominent countries with obese women, while Madagascar (1.1%) had the minimum obese women. Body mass index was significantly associated with hypertension and anemia. The behavioural or modifiable factors of hypertension and body mass index were; smoking, fruits, vegetables and alcohol consumption. While the non-modifiable significant factors include; age, residence, religion, education, wealth index, marital status, employment and number of children ever born. However, anemia shared similar factors except that smoking and vegetable consumption were not statistically significant. In addition, involvement in exercise was associated with anemia and hypertension.

**Conclusion:**

The problem of NCDs and associated factors remains high among women of reproductive age in sub-Sahara region. The findings of this study suggest that promotion of regular positive health care-seeking behaviour, screening and early treatment are essential to mitigate the burden of NCDs. Furthermore, preventive interventions of NCDs risk factors should be strengthened among key population through behavior change communication with support from government and stakeholders in health care.

## Background

Non-communicable diseases (NCDs) have recently become a prominent cause of mortality, which accounts for nearly two-third of deaths worldwide, while more than three-quarters of NCD-related mortality occur in resource-constrained settings [1]. In sub-Saharan Africa (SSA), the problem of NCDs and their risk factors are on the increase with several causes which may emerge in individuals as anemia, increased blood lipids and obesity, raised blood glucose, elevated blood pressure amongst others [2–4]. These risk factors have been associated with increased disability or morbidity and mortality. Healthy lifestyles and NCD control were the goals of Global Ministerial Conference, which prepared the platform for the United Nations (UN) Summit on NCD and subsequently, the declaration later in the year 2011 [5]. During the Moscow Declaration on NCDs in 2011, that proceeded from the ministerial conference, encompassed a responsibility from governments to create public policies that give equitable health supporting conditions that make communities, families and individuals to have healthy behaviors, as they give precedence to NCD prevention and control, maintaining other health care objectives and to reinforced the commitment of multiple sectors to address NCDs risk factors such as hypertension, anemia, overweight and obesity.

Obesity and overweight are prominent determinants of metabolic disorders including hypertension, diabetes, cancer and cardiovascular diseases [6–8]. An increase in overweight and obesity due to sedentary lifestyles or physical inactivity and feeding pattern specifically highly processed diet has affected community health in an unprecedented manner, by contributing to the risk of metabolic and cardiovascular diseases [9]. Similarly, several social, economic and demographic changes have occurred in resource-constrained settings; such as industrialization and urbanization which has influenced the lifestyles of people in developing countries in the past few decades. Obesity and overweight are major public health issues, particularly among women [4]. The effects of obesity and overweight on the sexual and reproductive life of women are more critical, especially during pregnancy. Sharp changes have been observed in many countries in Africa which are currently confronted with obesity and overweight among women, together with large increase in the occurrence of NCDs [10]. Evidence has revealed that women had approximately double the obesity prevalence of men [4]. Consequently, the problem of obesity and overweight among women is increasing at a rapid rate in developing countries including sub-Saharan Africa region. However, in multi-country studies, the prevalence of overweight and obesity is higher in urban areas than in rural areas of developing countries [4, 7].

Over the past decades, the prevalence of hypertension has been on the rise, from estimated 600 million in 1980 to approximately 1 billion in 2010 due to population growth, ageing and lifestyle [11]. The problem of hypertension and other prevalent NCDs has continued unabated in recent years, and it is the key risk factor for numerous cardiovascular diseases [2, 12]. Recent study reported that the prevalence of hypertension in African most populous country formed a crucial proportion of the total burden in Africa due to large population presently estimated over 170 million [2]. Considering a region with constrained health-care resources, the dynamic disease burden brings issues for health care providers, policy makers and progress toward achieving developmental goals [10]. Hypertension in sub-Saharan Africa has been associated with numerous factors including urbanization, lifestyles, diets, socio-economic status and physical inactivity [13–16].

NCDs is attributable to numerous risk factors including anemia, which is a prominent nutritional deficiency problem affecting women [17], and a risk factor for poor pregnancy outcome leading to problems that affect the life of foetus and mother alike [17, 18]. Anemia is an age-long public-health problem, particularly among the disadvantaged population in developing countries [19–21]. The prevalence of anemia is a vital health indicator for the measurements of maternal health outcomes [20]. Study on the magnitude of anemia and its determinants in vulnerable groups, such as women of reproductive age is essential for evidence-based intervention modalities, particularly in developing countries, where women suffer from micronutrient deficiencies and shortage of food [21]. The majority of women in resource-constrained settings are at higher risk of anemia due to increased level of macronutrient deficiencies, dietary iron deficiency and infections such as malaria and hookworm infestation amongst others [22]. Anemia has remained more prevalent among women in developing countries [3], while lactating mothers are most prone to blood loss during childbirth and maternal iron depletion [21].

Based on the worrisome prevalence in hypertension, anemia, overweight and obesity, and the possible increase in chronic diseases in sub-Saharan Africa countries, data on the correlates of the risk factors of NCDs is crucial and urgently needed to inform policy and programme interventions to address the problem in Africa. Evidence-based information on the prevalence and correlates of hypertension, anemia, overweight and obesity is paramount to understand the size of the problem, identify key population and develop effective intervention. Notably, a number of studies that examined hypertension, anemia, overweight and obesity prevalence in Africa centered on single country. However, the present study explored the prevalence and correlates of metabolic risk factors of NCDs in several sub-Saharan Africa countries and organized the results to aid understanding.

## Methods

### Data extraction

Data extracted for this study involved women of reproductive age (15–49 years) in nationally representative Demographic and Health Surveys conducted in 33 countries in sub-Sahara Africa region, 2008–2016. The study involved the following countries; Benin, Burkina-Faso, Burundi, Cameroon, Chad, Comoros, Congo, Cote d’Ivoire, Democratic Republic of Congo, Ethiopia, Gabon, Gambia, Ghana, Guinea, Kenya, Lesotho, Liberia, Madagascar, Malawi, Mali, Mozambique, Namibia, Niger, Nigeria, Rwanda, Sao Tome & Principe, Senegal, Sierra Leone, Tanzania, Togo, Uganda, Zambia and Zimbabwe (See details in Table 1). DHS data is publicly available and can be accessed from MEASURE DHS database at http://dhsprogram.com/data/available-datasets.cfm. DHS are usually implemented by the National Population Commission (NPC) with financial and technical assistance by ICF International provisioned through the USAID-funded MEASURE DHS program. DHS involved multi-stage stratified cluster design based on a list of enumeration areas (EAs), which are systematically selected units from localities and constitute the Local Government Areas (LGAs). Details of the sampling procedure have been reported in previously [23].

**Table 1:** Weighted percentage of high blood pressure, anemia and body mass index by countries in sub-Saharan Africa

Country	Survey year	Number of women (n)	High blood pressure (%)	Anemia (%)	Body mass index		
					Underweight (%)	Overweight (%)	Obese (%)
Benin	2012	16,599	4.4	41.4	6.0	20.3	7.2
Burkina-Faso	2010	17,087		48.8	14.8	8.2	2.9
Burundi	2016–17	17,269	1.0	39.3	17.7	6.6	1.8
Cameroon	2011	15,426		39.5	6.7	22.1	10.6
Chad	2014–15	17,719			18.0	9.6	2.3
Comoros	2012	5329			6.6	25.4	12.3
Congo	2012	10,819		54.2	13.6	17.6	8.5
Cote d’Ivoire	2012	10,060		53.7	7.1	19.3	6.6
Democratic Republic of Congo	2013–14	18,827		38.4	13.2	13.0	3.3
Ethiopia	2016	15,683		23.6	21.1	6.3	1.5
Gabon	2012	8422		60.6	6.7	26.1	18.9
Gambia	2013	10,233		59.0	15.7	16.1	7.3
Ghana	2014	9396	5.2	42.4	5.9	25.1	15.6
Guinea	2012	9142		49.1	11.5	14.9	4.6
Kenya	2014	31,079	9.4		8.6	22.7	10.1
Lesotho	2014	6621	17.3	27.3	4.1	25.5	19.9
Liberia	2013	9239			7.0	18.6	8.2
Madagascar	2009	17,375		35.3	25.5	5.2	1.1
Malawi	2015–16	16,592		32.7	6.7	16.1	5.4
Mali	2013	10,424		51.4	10.8	12.6	5.0
Mozambique	2011	13,537		54.0	7.9	12.7	4.0
Namibia	2013	1018		20.7	13.1	19.0	13.0
Niger	2012	11,160		45.8	13.9	13.5	3.7
Nigeria	2013	38,948			10.6	17.6	7.6
Rwanda	2014–15	13,497		19.2	6.1	18.4	3.9
Sao Tome & Principe	2009	2615		43.1	7.2	23.2	11.8
Senegal	2011	15,688		54.3	20.8	15.4	5.8
Sierra Leone	2013	16,658		44.8	8.6	13.9	4.9
Tanzania	2015–16	13,266		44.8	8.7	18.9	9.9
Togo	2013–14	9480		48.1	6.5	20.3	11.1
Uganda	2016	18,506		31.7	8.1	17.2	7.2
Zambia	2013–14	16,411			9.6	16.6	6.7
Zimbabwe	2015	9955		26.8	5.8	23.4	12.5

DHS program was established by the United States Agency for International Development (USAID) in 1984. It was designed as a follow-up to the World Fertility Survey and the Contraceptive Prevalence Survey projects. It was first awarded in 1984 to Westinghouse Health Systems (which subsequently evolved into part of OCR Macro). The project has been implemented in overlapping five-year phases; DHS-I ran from 1984 to1990; DHS-II from 1988 to1993; and DHS-III from 1992 to1998. In 1997, DHS was folded into the new multi-project MEASURE program as MEASURE DHS+. Since 1984, more than 130 nationally representative household-based surveys have been completed under the DHS project in about 70 countries. Many of the countries have conducted multiple DHS to establish trend data that enable them to gauge progress in their programs. Countries that participate in the DHS program are primarily countries that receive USAID assistance; however, several non-USAID supported countries have participated with funding from other donors such as UNICEF, UNFPA or the World Bank. DHS are designed to collect data on fertility and reproductive health, child health, family planning and HIV/AIDS. Due to the subject matter of the survey, women of reproductive age (15–49) are the main focus of the survey. Women eligible for an individual interview are identified through the households selected in the sample. Therefore, all DHS surveys utilize a minimum of two questionnaires-a Household Questionnaire and a Women’s Questionnaire.

### Measurement of outcome variable

The risk factors of NCDs considered in this study; were anemia, hypertension and BMI (underweight, overweight and obesity). DHS grouped non-pregnant “anemic” women at: Hb level < 12.0 g/dl and non-pregnant “not anemic” women at: Hb level ≥ 12.0 g/dl. To adjust for anemia during pregnancy, women who were pregnant at the time of the surveys were categorized as “anemic” at: Hb level < 11.0 g/dl and “not anemic” at: Hb level ≥ 11.0 g/dl [6, 17]. BMI was calculated as the ratio of weight in kilograms (kg) to the square of height in meters (m^2^). BMI was categorized into; underweight (BMI < 18.5 kg/m^2^), normal (BMI 18.5–24.9 kg/m^2^), overweight (BMI 25.0–29.9 kg/m^2^) and obesity (BMI ≥30 kg/m^2^) [6, 9]. Hypertension was defined as systolic blood pressure (SBP) ≥140 mmHg and/or diastolic blood pressure (DBP) ≥90 mmHg [6, 10]. The systolic and diastolic blood pressures were confirmed by repeating the measurements three times and taking the average.

### Independent variables

Several factors including socio-demographic, economic and other maternal determinants were measured in this study: age (years) of women were categorized to 15–19/20–24/25–29/30–34/35–39/40–44/45–49; place of residence: rural/urban; educational attainment: no formal education/primary/secondary/higher education; religious background: Christianity/Islam/other religion or no religion; marital status: never married/married or living with partner; currently working: yes/no; number of children ever born: nulliparous/1–4/> 4; alcohol consumption: yes/no; smoking: yes/no; fruits consumption: low (< 2 days/week)/moderate (2–3 days/week)/high (≥4 days/week); vegetable consumption: low (< 2 days/week)/moderate (2–3 days/week)/high (≥4 days/week). In addition, the wealth scores were measured using principal components analysis approach based on a list of household assets, which include, number of household members, wall and roof materials, floor types, access to potable water and sanitation, type of cooking fuel, ownership of television, radio, motorcycle, refrigerator amongst others. Based on the weighted wealth scores, households were grouped into wealth quintiles; poorest, poorer, middle, richer and richest. The computation of wealth scores variable was conducted by DHS and has previously been reported [24].

### Ethical considerations

We did the analyses using publicly available data from demographic health surveys. Ethical procedures were the responsibility of the institutions that commissioned, funded, or managed the surveys. All DHS surveys are approved by ICF international as well as an Institutional Review Board (IRB) in respective country to ensure that the protocols are in compliance with the U.S. Department of Health and Human Services regulations for the protection of human subjects.

### Statistical analyses

Prevalence of the risk factors of NCDs was reported by percentages. To adjust for data representation, the survey module (svyset) was used for all analyses to account for sample weight. Correlation matrix was used to conduct multicollinearity diagnostics to examine association between explanatory variables using a cut-off minimum of 0.6 known to cause multicollinearity [25]. All explanatory variables were retained for analysis due to lack of collinearity. Furthermore, variables that were statistically significant in the unadjusted model were added in the multivariable regression models to adjust for the effect of confounding. Binary and multinomial logistic regression models were used to investigate the factors associated with anemia, hypertension [14] and body mass index [4]. The level of statistical significance was set at 5%. All data analyses were conducted using Stata 13.0 (Statacorp, College Station, Texas, United States of America).

## Results

The description of high blood pressure (HBP), anemia and anomalous body mass index (BMI) by countries in sub-Saharan Africa was presented in Table 1. Notably, only 5 countries in sub-Saharan Africa region reported data on blood pressure, which include; Benin, Burundi, Ghana, Kenya and Lesotho. The percentage of HBP was highest among women in Lesotho with about 17.3% and lowest among women in Burundi (1.0%). More so, the results showed that anemia is prevalent among sub-Saharan Africa women; where more than half of the women from Gabon (60.6%), Gambia (59.0%), Senegal (54.3%), Congo (54.2%), Mozambique (54.0%), Cote d’Ivoire (53.7%) and Mali (51.4%) were reportedly anemic. However, Rwanda (19.2), Namibia (20.7%) and Ethiopia (23.6%) had the least anemic women. Further, Madagascar (25.5%), Ethiopia (21.1%), Senegal (20.8%), Chad (18.0%) and Burundi (17.7%) reported the highest underweight women, while Lesotho (4.1%) had the lowest percentage of underweight women. In addition, Gabon (26.1%), Comoros (25.4%), Ghana (25.1%), Zimbabwe (23.4%), Sao Tome & Principe (23.2%), Kenya (22.7%), Cameroon (22.1%) and Benin (20.3%) had the leading overweight women and Madagascar (5.2%) had the minimum overweight women in sub-Saharan Africa countries. The percentage of obesity in sub-Saharan Africa showed that Lesotho (19.9%), Gabon (18.9%) and Ghana (15.6%) were the prominent countries with obese women, while Madagascar (1.1%) had the minimum obese women (See details in Table 1).

The results from Table 2 show the distribution of women’s characteristics by the risk factors of noncommunicable diseases. High blood pressure increased with advances in age. Similarly, overweight and obesity was higher among older respondents. Compared with rural women, the urban women had higher proportion of high blood pressure (7.2% vs 4.5%), overweight (21.7% vs 12.9%) and obesity (11.8% vs 3.9%). The converse was true for the proportion of women with anemia and underweight. Furthermore, the pattern of the risk factors of noncommunicable diseases was similar across socioeconomic characteristics. Women with higher education and higher wealth index had higher proportion of high blood pressure, overweight and obesity. The converse was however true among women with anemia and underweight. The details of women’s characteristics are presented in Table 2.

**Table 2:** The distribution of respondents’ characteristics

Variable	n (%)	High blood pressure (%)	Anemia (%)	Body mass index (%)			
				Normal (%)	Underweight	Overweight	Obesity
Age							
15–19	99,944 (21.2)	1.3	41.5	72.8	18.6	7.5	1.1
20–24	86,128 (18.3)	3.0	41.4	73.1	10.7	13.1	3.1
25–29	82,221 (17.5)	4.6	40.6	66.9	9.2	17.7	6.2
30–34	67,361 (14.3)	5.5	40.2	61.8	8.6	20.3	9.4
35–39	56,964 (12.1)	7.5	41.9	58.3	8.8	21.4	11.5
40–44	42,935 (9.1)	10.6	42.6	55.7	9.5	21.8	13.0
45–49	34,863 (7.4)	14.1	40.4	54.9	10.2	21.7	13.2
Residence							
Urban	171,897 (36.5)	7.2	39.9	57.8	8.6	21.7	11.8
Rural	299,361 (63.5)	4.5	41.8	70.1	13.1	12.9	3.9
Educational level							
None	154,399 (32.8)	3.4	46.8	69.3	14.1	12.6	3.9
Primary	157,612 (33.4)	6.1	39.2	66.4	11.0	16.2	6.4
Secondary	139,247 (29.6)	5.8	38.5	62.7	9.8	18.5	9.0
Higher	19,945 (4.2)	9.4	30.8	49.3	5.9	27.1	17.7
Religion							
Christianity	292,827 (65.6)	5.6	37.2	65.3	10.0	17.2	7.4
Islam	127,841 (28.7)	5.0	48.3	65.6	14.5	14.3	5.6
Others/no religion	25,420 (5.7)	4.0	44.0	69.2	13.1	12.8	4.8
Marital status							
Never married	122,194 (25.9)	2.1	38.5	69.8	16.3	10.9	3.1
Married/currently living with a partner	349,063 (74.1)	6.9	42.0	64.0	9.8	18.0	8.2
Wealth index							
Poorest	93,862 (19.9)	3.3	45.5	72.2	16.3	9.5	2.1
Poorer	88,301 (18.7)	4.9	43.4	71.3	12.8	12.5	3.3
Middle	88,733 (18.8)	5.4	42.0	68.8	11.2	15.0	5.0
Richer	93,190 (19.8)	6.6	39.7	63.0	9.5	19.2	8.3
Richest	107,172 (22.7)	7.0	36.3	54.5	7.9	23.4	14.2
Working							
Not currently	187,389 (41.3)	5.1	41.9	67.0	14.1	13.8	5.1
Currently working	266,513 (58.7)	5.6	40.6	64.4	9.4	18.0	8.2
Number of children							
Nulliparous	124,666 (26.5)	1.8	39.5	69.6	16.4	11.1	2.9
1–4	224,224 (47.7)	6.9	40.9	64.4	9.3	18.0	8.3
4+	121,526 (25.8)	7.0	43.6	63.6	10.3	18.1	8.1
Alcohol consumption							
No	81,066 (76.1)	5.6	35.5	63.5	12.3	16.7	7.4
Yes	25,492 (23.9)	1.6	27.6	62.6	14.4	14.7	8.3
Currently smoke							
No	433,319 (97.6)	5.4	40.9	65.4	10.8	16.7	7.2
Yes	10,866 (2.4)	7.5	40.1	62.6	21.0	11.1	5.3
Exercise							
No	27,424 (80.4)	6.3	31.8	57.0	10.6	21.2	11.2
Yes	6692 (19.6)	9.3	26.5	57.5	11.1	19.9	11.5
Fruit consumption							
Low	7047 (38.4)	5.2	29.7	56.8	12.9	19.1	11.2
Moderate	5128 (27.9)	3.9	31.1	56.3	8.4	20.7	14.5
High	6174 (33.6)	4.8	33.6	50.9	5.5	25.2	18.5
Vegetable consumption							
Low	5405 (29.0)	5.5	31.3	55.8	11.5	20.2	12.4
Moderate	5156 (27.7)	3.7	31.9	55.9	9.7	20.9	13.5
High	8062 (43.3)	4.9	31.2	52.8	7.2	23.1	16.9

### Determinants of high blood pressure and anemia

The HBP regression model results applies only to 5 countries in sub-Saharan Africa region which include; Benin, Burundi, Ghana, Kenya and Lesotho where the data was captured. The results showed that women at higher age intervals were more likely to have HBP, compared with women aged 15–19 years. Rural women had 39% reduction in HBP, compared with women in the urban residence (OR = 0.61; 95%CI = 0.56–66). Educated women had higher odds of HBP, compared with women with no formal education. Women with no Christianity or Islamic background had 30% reduction in HBP, compared with women with Christianity background (OR = 0.70; 95%CI = 0.56–0.88). More so, women who are currently married or living with a partner were 3.39 times as likely to have HBP, compared with women who were never married (OR = 3.39; 95%CI = 3.02–3.81). Women with higher wealth index or those employed had increase in HBP, compared with poorest or unemployed women. Women who had ever given birth had significant increase in the odds of HBP, compared with women with no childbirth. Further, alcohol, fruit and vegetable consumption showed significant reduction in HBP. Meanwhile, involvement in smoking and exercise were more likely to be associated with HBP. Body mass index was significantly associated with HBP; underweight women had 34% reduction in HBP compared with women of normal body mass index (OR = 0.66; 95%CI = 0.54–0.82). Conversely, overweight women were 2.44 times as likely to have HBP, compared with women with normal body weight (OR = 2.44; 95%CI = 2.19–2.72); and obese women were 5.34 times as likely to have HBP compared with normal body mass index women (OR = 5.34; 95%CI = 4.75–5.99) (Details are shown in Table 3).

**Table 3:** Factors associated with high blood pressure and anemia

Variable	High blood pressure		Anemia	
	OR	95% CI	OR	95% CI
Age				
15–19	1.00		1.00	
20–24	2.32	1.88–2.87*	1.01	0.97-1.03
25–29	3.61	2.96–4.40*	0.96	0.94-0.99*
30–34	4.40	3.61–5.37*	0.95	0.92-0.98*
35–39	6.14	5.04–7.46*	1.02	0.98-1.05
40–44	8.95	7.37–10.87*	1.05	1.01-1.09*
45–49	12.45	10.25–15.12*	0.96	0.92-0.99*
Residence				
Urban	1.00		1.00	
Rural	0.61	0.56–0.66*	1.08	1.06-1.10*
Educational level				
None	1.00		1.00	
Primary	1.85	1.66–2.07*	0.73	0.72-0.75*
Secondary	1.76	1.57–1.97*	0.71	0.70-0.73*
Higher	2.96	2.51-3.48*	0.51	0.48-0.54*
Religion				
Christianity	1.00		1.00	
Islam	0.90	0.77–1.04	1.58	1.54–1.61*
Others/no religion	0.70	0.56–0.88*	1.33	1.28-1.38*
Marital status				
Never married	1.00		1.00	
Married/currently living with a partner	3.39	3.02–3.81*	1.16	1.13-1.18*
Wealth index				
Poorest	1.00		1.00	
Poorer	1.50	1.30–1.73*	0.92	0.89-0.94*
Middle	1.66	1.45-1.91*	0.87	0.84-0.89*
Richer	2.04	1.78-2.33*	0.79	0.77-0.81*
Richest	2.20	1.94-2.51*	0.68	0.66-0.70*
Currently working	1.11	1.02–1.20*	0.95	0.93-0.97*
Number of children				
Nulliparous	1.00		1.00	
1–4	4.02	3.53–4.57*	1.06	1.04-1.08*
4+	4.02	3.49-4.63*	1.18	1.15-1.21*
Alcohol consumption	0.27	0.22–0.32*	0.69	0.66-0.72*
Smoking	1.41	1.17-1.70*	0.97	0.92-1.02
Exercise	1.52	1.35–1.71*	0.77	0.68-0.87*
Fruit consumption				
Low	1.00		1.00	
Moderate	0.74	0.57–0.97*	1.07	0.96-1.19
High	0.91	0.72–1.14	1.20	1.08–1.33*
Vegetable consumption				
Low	1.00		1.00	
Moderate	0.66	0.51–0.86*	1.03	0.92-1.15
High	0.89	0.70–1.11	0.99	0.90–1.11
Body mass index				
Normal	1.00		1.00	
Underweight	0.66	0.54–0.82*	1.09	1.05-1.12*
Overweight	2.44	2.19-2.72*	0.84	0.82-0.86*
Obesity	5.34	4.75-5.99*	0.71	0.68-0.74*

^*^significant at *p* < 0.05; OR = Odds ratio

Also, Table 3 shows factors associated with anemia among women of reproductive age. The age of women was significantly associated with anemia among women. Furthermore, rural women were 1.08 times as likely to have anemia, compared with women in urban residence (OR = 1.08; 95%CI = 1.06–1.10). Educated women, those from households with higher wealth index or those currently working had significant reduction in the odds of anemia, compared with women without formal education, those from poorest households or the unemployed. The religious background of women was significantly associated with anemia. Married women or those living with a partner were 1.16 times as likely to have anemia, compared with unmarried women (OR = 1.16; 95%CI = 1.13–1.18). Childbirth was positively associated with anemia, compared with nulliparous women. Consumption of alcohol was significantly associated with reduction in anemia (OR = 0.69; 95%CI = 0.66–0.72), while involvement in exercise had 23% reduction in anemia, compared with women who do not exercise (OR = 0.77; 95%CI = 0.68–0.87). High consumption of fruit was positively associated with anemia. Underweight women were 1.09 times as likely to have anemia compared with women with normal body mass index (OR = 1.09; 95%CI = 1.05–1.12). Meanwhile, overweight and obese women had 16% (OR = 0.84; 95%CI = 0.82–0.86) and 29% (OR = 0.71; 95%CI = 0.68–0.74) reduction in the odds of anemia, compared with women with normal body mass index.

### Determinants of body mass index

Multinomial logistic regression model was used to obtain the relative-risk ratio estimates of the factors associated with underweight, overweight and obesity relative to normal body mass index. Results showed a decrease in relative risk ratios of higher age interval for underweight women relative to normal body mass index, compared with women aged 15–19 years. On the contrary, higher age categories were more likely to be overweight and obese relative to normal body mass index, compared with women aged 15–19 years. While rural women were 1.25 times as likely to be underweight, compared with urban women (RR = 1.25; 95%CI = 1.22–1.28); the rural dwellers had 51% (RR = 0.49; 95%CI = 0.48–0.50) and 72% (RR = 0.28; 95%CI = 0.27–0.29) reduction in overweight and obesity relative to women with normal body mass index, compared with women from urban residence. In addition, educated women, those from households with higher wealth index or employed had significant reduction in underweight, but had higher risk of overweight and obesity relative to women with normal body mass index, compared with women with no formal education, from poorest households or unemployed.

Respondent’s religious background was associated with body mass index. Furthermore, married women or those living with partners had 35% reduction in underweight, compared with those never married (RR = 0.65; 95%CI = 0.64–0.67); however, married women or those currently living with partners were 1.81 (RR = 1.81; 95%CI = 1.76–1.86) and 2.91 (RR = 2.91; 95%CI = 2.77–3.04) as likely to be overweight and obesity, compared with never married women. Parity was significantly associated with body mass index; women who had given birth had significant reduction in the risk of underweight, compared with nulliparous women; but women who had given birth had higher risk of overweight and obesity, compared with the nulliparous women relative to the normal body mass index. The consumption of alcohol was associated with body mass index. Also, involvement in smoking increased the risk of underweight (RR = 2.03; 95%CI = 1.92–2.16), but reduced the risk of overweight (RR = 0.70; 95%CI = 0.65–0.75) and obesity (RR = 0.78; 95%CI = 0.70–0.86). Moderate and high fruits and vegetable consumption, lower the risk of underweight, but increased the risk of overweight and obesity, compared with low consumption level relative to normal body mass index women. Details are presented in Table 4.

**Table 4:** Factors associated with underweight, overweight and obesity

Variable	Underweight		Overweight		Obesity	
	RR	95% CI	RR	95% CI	RR	95% CI
Age						
15–19	1.00		1.00		1.00	
20–24	0.57	0.55–0.59*	1.73	1.66–1.80*	2.71	2.47–2.96*
25–29	0.53	0.52–0.56*	2.57	2.47–2.67*	5.94	5.46–6.46*
30–34	0.54	0.52–0.56*	3.19	3.06–3.31*	9.78	9.01–10.62*
35–39	0.59	0.57–0.62*	3.56	3.42–3.70*	12.72	11.72–13.82*
40–44	0.66	0.63–0.70*	3.80	3.64–3.97*	15.00	13.80–16.31*
45–49	0.73	0.69–0.76*	3.84	3.67–4.02*	15.45	14.18–16.83*
Residence						
Urban	1.00		1.00		1.00	
Rural	1.25	1.22–1.28*	0.49	0.48–0.50*	0.28	0.27–0.29*
Educational level						
None	1.00		1.00		1.00	
Primary	0.82	0.79–0.84*	1.34	1.30–1.37*	1.69	1.63–1.77*
Secondary	0.77	0.75–0.79*	1.62	1.58–1.66*	2.52	2.42–2.63*
Higher	0.59	0.55–0.64*	3.02	2.88–3.16*	6.34	5.98–6.72*
Religion						
Christianity	1.00		1.00		1.00	
Islam	1.44	1.41–1.48*	0.83	0.81–0.85*	0.75	0.73–0.78*
Others/no religion	1.24	1.18–1.30*	0.70	0.67–0.74*	0.61	0.56–0.66*
Marital status						
Never married	1.00		1.00		1.00	
Married/currently living with a partner	0.65	0.64–0.67*	1.81	1.76–1.86*	2.91	2.77–3.04*
Wealth index						
Poorest	1.00		1.00		1.00	
Poorer	0.79	0.77–0.82*	1.34	1.29–1.39*	1.64	1.52–1.76*
Middle	0.72	0.69–0.75*	1.66	1.60–1.73*	2.55	2.38–2.73*
Richer	0.67	0.65–0.69*	2.33	2.25–2.41*	4.58	4.29–4.89*
Richest	0.64	0.62–0.67*	3.28	3.17–3.39*	9.09	8.54–9.68*
Currently working	0.69	0.68–0.71*	1.35	1.32–1.38*	1.69	1.64–1.75*
Number of children						
Nulliparous	1.00		1.00		1.00	
1–4	0.61	0.59–0.63*	1.76	1.71–1.81*	3.09	2.95–3.24*
4+	0.69	0.66–0.71*	1.79	1.74–1.84*	3.04	2.89–3.19*
Alcohol consumption	1.19	1.13–1.25*	0.89	0.85–0.94*	1.13	1.06–1.20*
Smoking	2.03	1.92–2.16*	0.70	0.65–0.75*	0.78	0.70–0.86*
Exercise	1.03	0.93–1.14	0.93	0.86–1.01	1.02	0.92–1.13
Fruit consumption						
Low	1.00		1.00		1.00	
Moderate	0.66	0.56–0.79*	1.09	0.96–1.25	1.30	1.12–1.52*
High	0.48	0.40–0.57*	1.47	1.31–1.66*	1.84	1.59–2.12*
Vegetable consumption						
Low	1.00		1.00		1.00	
Moderate	0.85	0.71–1.01	1.03	0.90–1.18	1.09	0.92–1.28
High	0.66	0.56–0.78*	1.21	1.07–1.37*	1.44	1.24–1.66*

*RR* Relative-risk ratio; *significant at *p* < 0.05;

## Discussion

In this study, we extensively explored the prevalence of prominent risk factors of NCDs which include hypertension, anemia, underweight, overweight and obesity among women of reproductive age in sub-Sahara Africa countries. Furthermore, we investigated the correlates of the risk factors of NCDs with the aim of understanding the pattern of the problem and how best to prevent and control it. The findings of this study revealed that a large number of women in sub-Sahara Africa countries have suffered from hypertension, anemia, underweight, overweight and obesity. The most striking findings in our study were the very high prevalence in hypertension, anemia, underweight, overweight and obesity among women of reproductive age in sub-Sahara Africa countries. These findings are similar to the reports from previous studies in developing countries [2, 4, 22, 26, 27].

This study revealed that living in rural residence, smoking and alcohol consumption were associated with reduction in the risk of overweight and obesity. Conversely, high consumption of fruits and vegetables were positively associated with overweight and obesity. In addition, higher number of children ever born, older age, involvement in marriage or living with a partner, women’s employment, high socio-economic status such as wealth index and education were also associated with higher risk of overweight and obesity. Our findings were consistent with the results of previous studies on the factors associated overweight and obesity [8, 9, 28]. The increase in the risk of overweight and obesity among women who are employed, have formal education and higher wealth index showed that as the economy and individuals income improve, they tend to have sedentary lifestyles, reducing their physical activity levels. Urbanization and adoption of Western lifestyles could also contribute to dietary shift to inappropriate food choices such as frequent intake of “fast foods” including sugar and fat leading to increase in body weight [29]. More so, the impact of marriage on overweight and obesity could be as a result of women’s sexual and reproductive activities such as child bearing with increase in sedentary lifestyles. The content of alcohol or “hot drinks” and nicotine obtain from smoking, especially for heavy users could be responsible for the breakdown of body cells leading to reduction in the risk of overweight and obesity as found in this study [30, 31]. Notwithstanding, smoking and alcohol consumption are known to have negative long-term health effect to the body.

Consumption of fruits, vegetables and rural residence were significantly associated with reduction in the odds of hypertension among women in sub-Saharan Africa. This findings is similar to previous report [32]. The factors associated with increase in the odds of hypertension were; high body mass index, smoking, large number of children ever born, high wealth index, marriage or living with a partner, education and older age. Similar findings have been made by previous studies in developing countries [10, 12, 26, 32]. Reduction in the risk of hypertension among rural dwellers could be due to differences in life styles such as dietary patterns and physical activity. Fruits and vegetables contain micronutrients and vitamins that aid metabolic activities of the body and also help in body circulatory system [33, 34]. More so, the association between older age and increased risk of hypertension could be due to the biological effect of increased arterial resistance as a result of arterial thickening as one grows older [35, 36]. In addition, our findings of overweight and obesity as factors of hypertension agree with the general view that adiposity is linked to hypertension [37–39]. The associations between hypertension and metabolic, socio-demographic, economic and behavioural factors revealed epidemiological transition in resource-constrained settings usually explained by economic development leading to urbanization with increased sedentary life style, deviations in dietary habits, social stress and smoking. Sub-Saharan Africa countries are likely to be undergoing similar development.

Socio-economic status of women was significantly associated with anemia among women. Women from rural residence, high number of children ever born, married and those from lower socio-economic class had higher odds of anemia. The findings of this study is consistent with previous results [17, 40]. Other studies have argued that the association between the place residence and health problems in developing countries is driven mainly by higher individual-level and community-level socioeconomic status. These findings could also be due to those women from lower socio-economic status lack the finance to purchase quality food as well as sufficient quantity of foods for proper feeding. The results also showed that fruits consumption does not help to cure anemia. However, high body mass index was associated with reduction in the odds of anemia among women in sub-Saharan Africa. It is possible the women with high body mass index are usually among the well-off and those in the advantaged group, who are able to afford quality and adequate food for proper feeding [17, 40].

### Strength and limitations

This study used nationally representative secondary data and the findings are generalizable for the women of reproductive age in sub-Saharan Africa countries. Sample size was considerably large that were collected from 33 countries in sub-Saharan Africa which allowed widespread reporting of the prevalence of the risk factors of non-communicable diseases such as hypertension, anemia, underweight, overweight and obesity. However, a major drawback is that cross-sectional study design used cannot adequately establish causality [41, 42]. More so, since the study utilized secondary data, we were unable to measure vital micronutrients such as vitamin B12, folate and vitamin A. Caution must be taken in the generalization of the findings on high blood pressure as only 5 countries in sub-Saharan Africa region reported on the data.

## Conclusion

The goal of this study was to explore the prevalence and correlates of the risk factors of NCDs in sub-Sahara Africa region. In presenting an evidence-based context for government and other health policy makers on the strategies to reduce this burden in low-resource settings, detailed up-to-date information on the metabolic risk factors of non-communicable diseases has been provided in order to match this with available resources. A major strategy to prevent or control the occurrence of NCDs is to focus on reducing anemia, hypertension, overweight and obesity.

Interventions that focus on the modifiable determinants of overweight and obesity can help to prevent the increasing burden of non-communicable disease in sub-Saharan Africa. Hence, future obesity prevention programmes in resource-constrained transitioning settings will need to foremost, consider the family level with a target on well-off women. Development of community-based programmes influencing dietary patterns, physical activity, alcohol use and smoking, behavior change communication about the effect of hypertension, anemia and high body mass index, could help to reduce chronic disease associated with the metabolic risk factors of non-communicable diseases. This will also involve a multi-dimensional strategy and will require intervention at the individual and population levels. Health policy makers and government need to focus on widespread interventions through community health extension programmes.

## Abbreviations

BMI: Body mass index
DHS: Demographic Health Survey
EAs: Enumeration areas
Hb: Hemoglobin
IRB: Institutional Review Board
NCDs: Non-communicable diseases
SSA: Sub-Saharan Africa
UN: United Nations
